# Outcomes Following Stem Cell-Based Therapies for Hip Osteoarthritis: A Scoping Review

**DOI:** 10.7759/cureus.96419

**Published:** 2025-11-09

**Authors:** Guilherme E. Reali, Daniel Araujo Fernandes, Eduardo Campos Martins

**Affiliations:** 1 Department of Surgery, Universidade Federal de Santa Catarina, Florianópolis, BRA; 2 Department of Hip Surgery, Hospital das Clínicas da Universidade de São Paulo, Instituto de Ortopedia e Traumatologia, São Paulo, BRA

**Keywords:** hip, osteoarthritis, pain, regenerative medicine, stem cells

## Abstract

Hip osteoarthritis (OA) is a prevalent and debilitating condition for which current treatment options remain limited, especially in early to moderate stages. Stem cell-based therapies, particularly those using mesenchymal stem cells (MSCs), have gained attention due to their potential regenerative and anti-inflammatory properties. This scoping review aimed to map and summarize current clinical evidence on the efficacy and safety of intra-articular stem cell therapies for hip OA.

This review followed the PRISMA-ScR guidelines and was registered on the Open Science Framework (DOI: 10.17605/OSF.IO/G8V9S). A systematic search of PubMed, Embase, Cochrane Library, Web of Science, and Scopus was conducted from December 2024 to January 2025. Inclusion criteria comprised clinical studies evaluating intra-articular stem cell-based therapies in adults with hip OA. Excluded were non-clinical studies, reviews, and studies involving joints other than the hip or non-stem cell interventions. Two reviewers independently selected studies and extracted data, resolving disagreements through discussion with a third reviewer.

Out of 286 records identified, 9 studies met the inclusion criteria. These included five cohort studies and four case series, assessing various MSC sources: bone marrow-derived MSCs (BM-MSCs), adipose-derived MSCs (AD-MSCs), stromal vascular fraction (SVF), and amniotic-derived preparations. Pain reduction was consistently observed, with average visual analog scale (VAS) score reductions of 30-50%. Functional improvement was reported via metrics like the Harris Hip Score and Western Ontario and McMaster Universities Osteoarthritis Index (WOMAC). Radiologic evidence of cartilage repair was limited and inconsistent. Adverse events were rare and mild, including transient joint discomfort and local swelling, with no major complications reported.

Stem cell-based therapies show promising short- to mid-term outcomes in pain relief and functional improvement for hip OA, with favorable safety profiles. However, heterogeneity in study designs and limited long-term structural outcomes highlight the need for standardized protocols and robust randomized trials to confirm regenerative efficacy.

## Introduction and background

Osteoarthritis (OA) is a highly prevalent condition with a significant global impact. It affects an estimated 7.6% of the world’s population, equivalent to 595 million people in 2020 [1]. Projections indicate that this burden will increase by 2050, placing additional strain on healthcare systems worldwide [1].

Hip OA, one of the most clinically relevant types of OA, is driven by pathological biomechanical stress. This stress disrupts the balance between joint tissue synthesis and degradation. As a result, cartilage wear occurs, characterized by microdamage, loss of type II collagen and proteoglycans, and increased release of pro-inflammatory mediators. Collectively, these changes weaken the articular cartilage and contribute to disease progression [2].

According to recent evidence, conservative care remains the first-line treatment for hip OA [3,4]. However, many patients eventually require total hip arthroplasty (THA) [3]. While THA often significantly improves pain and quality of life, a notable proportion of patients do not achieve satisfactory symptom relief [3,5]. Moreover, THA failure and its associated complications remain major challenges for orthopedic surgeons [6,7].

Given the limited self-repair capacity of articular cartilage, mesenchymal stem cells (MSCs) have received considerable attention as a potential therapy. MSCs can differentiate into chondrocytes and secrete bioactive molecules that support tissue repair and modulate inflammation [8-11]. Different MSC sources offer distinct clinical advantages. Bone marrow-derived MSCs (BM-MSCs) are the most studied, but their use is limited by invasive harvest procedures and low cell yield. Adipose-derived MSCs (AD-MSCs) are more accessible and abundant. Perinatal-derived MSCs, such as those from umbilical cord or placenta, provide immunoprivileged properties and high proliferative capacity, making them attractive for clinical use [10,11].

Ongoing clinical and preclinical studies continue to evaluate the efficacy and safety of MSC therapies. Researchers are exploring optimized isolation techniques, standardized protocols, and strategies to reduce variability in cell quality. In this context, our study aims to map and summarize the current evidence on stem cell therapies for hip OA. We specifically focus on their potential to reduce hip pain, improve joint function, and regenerate cartilage while delaying or preventing the need for THA.

## Review

Methods

Protocol and Registration

This scoping review was reported according to the Preferred Reporting Items for Systematic reviews and Meta-Analyses extension for Scoping Reviews (PRISMA-ScR) Checklist [12] (Appendix). The protocol of this study was registered on the Open Science Framework registration platform (https://osf.io/) under the identification code DOI: 10.17605/OSF.IO/G8V9S.

Eligibility Criteria

We included clinical trials and observational studies that assessed the use of stem cell-based intra-articular injections for the treatment of hip OA in adult patients. Studies were considered regardless of the period of publication or language restrictions.

Any kind of association or therapeutic outcome was considered, including the efficacy, safety, and comparative effectiveness of different stem cell therapies, their impact on pain reduction, functional improvement, and cartilage regeneration.

Studies meeting the following criteria were included: adults (≥18 years old) diagnosed with hip OA, based on any reliable and valid diagnostic criteria. The intervention encompassed studies evaluating stem cell-based intra-articular injections, including bone marrow-derived mesenchymal stem cells (BM-MSCs), adipose-derived mesenchymal stem cells (AD-MSCs), stromal vascular fraction (SVF), umbilical cord-derived stem cells, amniotic-derived stem cells, and other orthobiologic treatments explicitly involving stem cells. Eligible study designs included clinical trials (randomized or non-randomized) and observational studies, such as prospective and retrospective cohort studies, case-control studies, and cross-sectional studies. Outcome measures included studies assessing one or more of the following: pain reduction, functional improvement, cartilage regeneration, safety and adverse effects, delay in disease progression, and biomarkers of cartilage repair.

Studies focusing only on knee OA or other joints were excluded. Additionally, studies evaluating non-stem cell-based cell therapies, such as platelet-rich plasma (PRP), plasma-derived products, growth factor injections alone, and exosome-only therapies were not considered. We did not include animal or in vitro studies, narrative reviews, systematic reviews, meta-analyses, or pilot studies. The study selection and data extraction processes were independently conducted by two reviewers. Any discrepancies or disagreements between the reviewers were resolved through discussion and consensus, with the help of a third reviewer, ensuring methodological consistency and minimizing selection bias.

Search Strategies

Supported by PRISMA-ScR recommendations and its main article citations [12], our search strategies were developed based on multiple blinded tests conducted by the first and senior authors, with the assistance of an experienced health science librarian. In these tests, we gradually increased the specificity of the search keys through the cautious addition of equivalent and synonymous terms. Crossing the results of each search key with progressively higher specificity allowed us to identify the key with approximately the best specificity, without compromising sensitivity. Additionally, we employed an artificial intelligence software (OpenEvidence, Cambridge, MA) to complement our tests on maintaining the sensitivity of the search strategies, providing additional evidence that the search keys were appropriate. Search strategies were applied to five major databases: PubMed, Embase, Cochrane Library, Web of Science, and Scopus. The searches were conducted between December 15, 2024, and January 29, 2025. All references were imported into the Rayyan software for an accurate review management, and duplicate records were removed. The search strategies applied in the databases can be found in Appendix 1.

Data Extraction

Two reviewers independently extracted the data in duplicate, and disagreements were resolved through discussion and consensus, with input from a third reviewer when necessary. This process ensured accuracy and minimized bias in data handling. Data were extracted using a standardized form developed a priori, capturing study characteristics, patient demographics, interventions, comparators (if any), outcomes, and follow-up details. Consistent with the Joanna Briggs Institute methodology for scoping reviews [13], a formal quality assessment or risk of bias appraisal of the included studies was not undertaken, as our aim was to map the extent and nature of the available evidence rather than to assess study quality in detail.

Results

**Figure 1:**
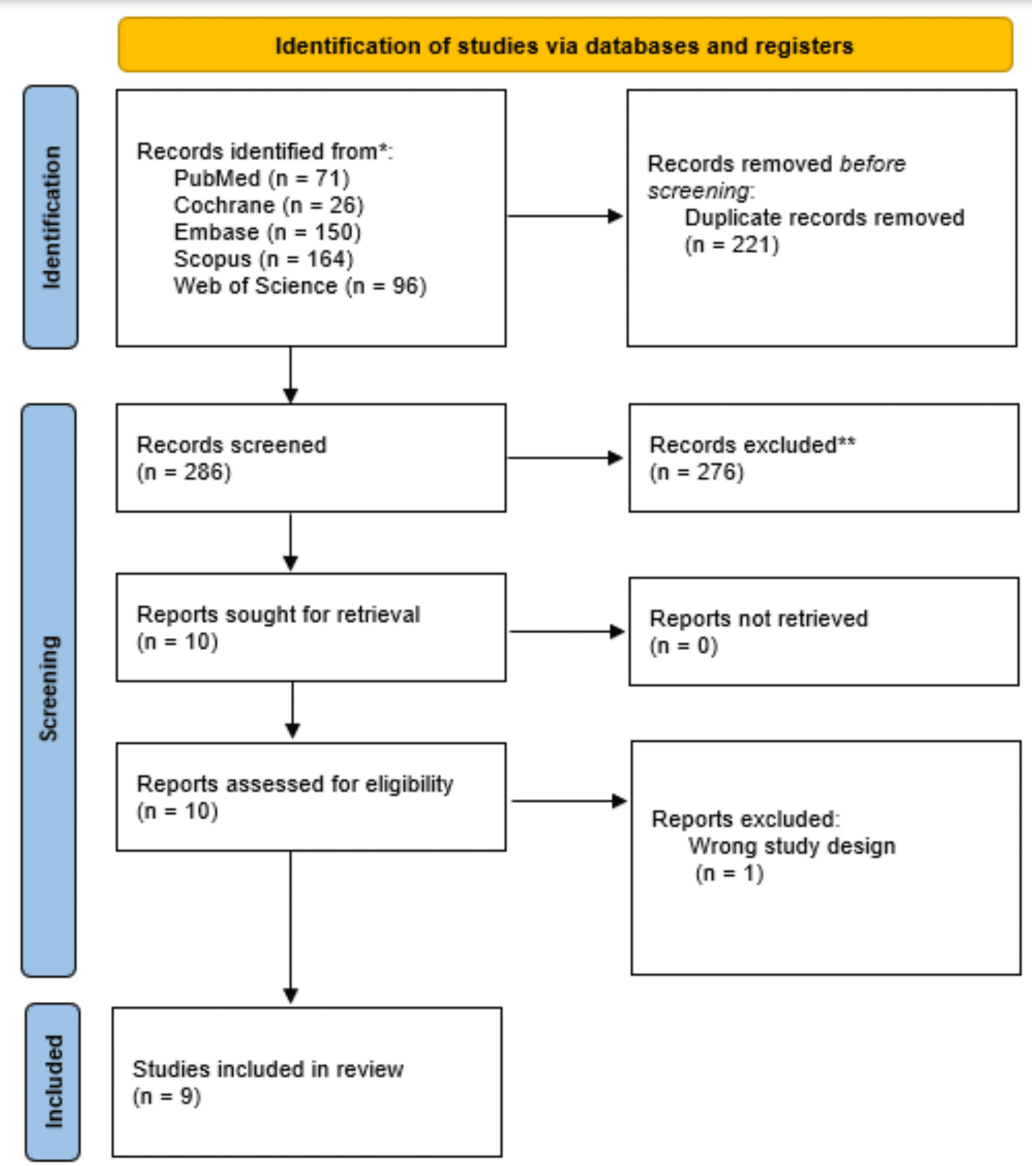
PRISMA flowchart diagram of study selection

A total of 507 articles were identified across the databases, as shown in the PRISMA flowchart (Figure 1). After the removal of 221 duplicate records, 286 studies were screened based on title and abstract. A complete reading of 10 articles was conducted; however, 1 was excluded because it was a pilot study [14].

Of the nine included studies, five were cohort studies with defined inclusion and exclusion criteria, exploring associations between interventions and outcomes [15-19]. Four studies were case series providing individual-level data without hypothesis testing for cause and effect [20-23]. The studies originated from various countries: three from Italy, two from the United States, two from Canada, one from Japan, and one from Chile.

Sample sizes ranged from 6 to 96 participants, with an average of approximately 30 participants per study. Participant ages ranged from 47 to 67 years. Most studies focused on mild to moderate hip osteoarthritis, as diagnosed using radiographic grading systems like Kellgren-Lawrence (KL) or Tonnis classification [15-17,19].

All studies evaluated stem cell-based intra-articular injections, categorized as bone marrow-derived BM-MSCs [15,17-20], AD-MSCs and SVF [16,21,23], and amniotic suspension allografts containing stem cell components [15]. Injection protocols varied among studies, with four studies employing single injections [15,17,18,20], while the remaining studies used multiple injections over specific intervals [16,21-23]. Cell dosages ranged from 5 × 10⁵ to 3 × 10⁷ cells per injection, with injection volumes of 5-20 milliliters. Regarding the procedural approach, all studies performed injections via image-guided intra-articular delivery (fluoroscopy or ultrasound), with no study reporting the use of decompression techniques. Tables 1-4 provide detailed information on the methodologies used in each study.

**Table 1:** Study identification, objectives, and population of interest OA: osteoarthritis; ASA: amniotic suspension allograft; SVF: stromal vascular fraction; BM-MSCs: bone marrow-derived mesenchymal stem cells; AD-MSCs: adipose-derived mesenchymal stem cells; MFAT: microfragmented adipose tissue; PRP: platelet-rich plasma; C-PRP: umbilical cord platelet-rich plasma; A-PRP: autologous platelet-rich plasma; BMC: bone marrow concentrate; BMI: body mass index; VAS: visual analog scale; NSAIDs: nonsteroidal anti-inflammatory drugs; MRI: magnetic resonance imaging

Study		Population		
Author, Year, Country, Design	Objective	Sample Sex (M/F) Mean age or range	Inclusion criteria	Exclusion criteria
Meadows et al. [15], 2022, United States, Prospective Case Series	To examine the effects of a commercially available ASA in patients with moderate hip OA. To evaluate the safety and effectiveness of ASA in pain relief and functional improvement.	Total sample: 10 patients. Sex: 5 males, 4 females (1 patient withdrew from the study). Age range: 47-67 years (mean 54.2 ± 6.0 years)	Adults aged 18-70 years with clinical and radiographic evidence of hip OA. Tonnis grade 1 or 2 OA based on radiographic assessment. Minimum Tegner activity scale score of 2. Pain score ≥ 4 on a 7-day average pain scale. BMI < 40. Female participants had to use contraception, be menopausal, or have surgical sterilization.	History of diabetes, rheumatoid arthritis, autoimmune disorders, malignancy (last 5 years), or severe obesity (BMI > 40). Use of NSAIDs or pain medications within 15 days before injection. Use of corticosteroid injections or viscosupplements within the last 6 months. History of hip dysplasia (center-edge angle < 25° or Tonnis angle > 10°). Recent hip surgery (past 6 months).
Onoi et al. [16], 2023, Japan, Prospective Case Series	To evaluate the short-term clinical outcomes of adipose-derived SVF cell therapy for hip OA.	Total sample: 42 patients. Sex: 37 females, 5 males Mean age: 60.2 ± 9.4 years	Patients of any age diagnosed with hip OA. Considerable pain and functional decline. Failure of conservative treatments such as physical therapy, pharmacotherapy, and intra-articular injections of hyaluronic acid or steroids. Provided written informed consent.	Severe bone loss or dislocation observed on preoperative radiographs. History of hip injury requiring surgery. Active or previous infection of the hip joint. History of severe systemic diseases (e.g., inflammatory conditions or vascular disorders).
Heidari et al. [21], 2022, United Kingdom, Observational, Intention-to-treat Comparative	To compare the effects of MFAT and a combination of MFAT with PRP for the treatment of hip OA, evaluating pain reduction and functional improvement.	Total sample: 147 patients, MFAT group: 57 patients. MFAT + PRP group: 90 patients. Sex: Not explicitly stated. Mean age: 60 years for both groups.	Patients diagnosed with hip OA based on X-ray and/or MRI.	Recent hip injury (<3 months). Malignancy. Infectious joint disease. Anticoagulation therapy. Pregnancy or thrombocytopenia. Coagulation disorders. Intra-articular steroid injection within the last three months.
Rodriguez-Fontan et al. [22], 2018, United States, Prospective Cohort	To assess the clinical outcomes, satisfaction, and safety of patients undergoing intra-articular injection of BM-MSCs for the treatment of early knee and hip OA.	Total sample: 19 patients. Joints treated: 25 (10 knees, 15 hips). Sex: 16 females, 3 males. Mean age: 58 ± 12.7 years (range 30-80 years)	Patients aged 18 years or older. Diagnosed with early OA of the knee (Kellgren-Lawrence grade I-II) or hip (Tönnis grade I-II). Failed conservative treatments, including physical therapy and NSAIDs, for at least six months.	Pregnancy. Malignancy. Rheumatologic diseases. Infection. Advanced OA (Kellgren-Lawrence grade III-IV, Tönnis grade III). Prior intra-articular steroid injections or joint surgery.
Natali et al. [17], 2023, Italy, Prospective Case Series	To evaluate the clinical effectiveness of intra-articular injections of autologous MFAT for hip OA over a three-year period.	Total sample: 55 patients (initially 71, but 16 were lost to follow-up). Sex: 22 males, 33 females. Mean age: 52.5 years (standard deviation ±10.9).	Patients with hip OA who experienced pain resistance to NSAIDs for at least four months, functional limitations, or failure of prior conservative treatments.	History of trauma to the symptomatic hip.
Mardones et al. [23], 2017, Chile, Prospective Cohort Study	To investigate the safety and efficacy of intra-articular infusion of ex vivo expanded autologous BM-MSCs in patients with hip OA.	Sample, sex, and age: Total sample: 10 patients. Sex: Not specified. Mean age: 60 years or older	Patients aged 60 years or older with radiographic evidence of mild to moderate hip OA. Patients with pain refractory to analgesics, hyaluronic acid, or corticosteroid injections, with a VAS pain score of 40 or higher.	Intra-articular space less than 1 mm. Severe cartilage loss as measured by MRI. Failure to complete the required number of cell infusions.
Dall’Oca et al. [18], 2019, Retrospective Case Series	To assess the feasibility and effectiveness of intra-articular injections of AD-MSCs in the treatment of hip OA.	Sample, sex, and age: Total sample: 6 patients. Sex: 5 males, 1 female. Mean age: 52 years (range 37 to 60 years).	Patients with persistent hip pain resistant to NSAIDs for at least six months. Functional limitations and failure of prior conservative treatments.	Recent trauma to the symptomatic hip. High-grade OA (greater than grade 2 on the Tonnis classification system).
Burnham et al. [19], 2021, Canada, Prospective Cohort	To assess the safety and effectiveness of a single intra-articular BMC injection for the treatment of knee and hip OA in a Canadian cohort.	Total sample: 112 patients. Joints treated: 82 knees, 20 hips, 10 both knees and hips. Sex: 57% male. Mean age: 64.1 ± 9.1 years (range 42–93 years).	Patients with refractory knee and/or hip OA. Clinical and radiographic evidence of OA (Kellgren-Lawrence grades 1–4). Completed 3- and 6-month follow-up assessments.	Inflammatory arthritis. Generalized or local infection at the bone marrow donor site or recipient joint. Allergy to local anesthetics. Intra-articular corticosteroid injection within the prior two months. Current use of oral NSAIDs or prednisone.
Mazzotta et al. [20], 2022 Italy, Observational, Intention-to-Treat Comparative	To compare the safety and clinical efficacy of intra-articular injections of C-PRP versus A-PRP in the treatment of hip OA.	Total sample: 96 patients (50 in the A-PRP group, 46 in the C-PRP group). Sex: 60 males, 36 females. Mean age: 47.1 ± 11.9 years in the C-PRP group, 49.5 ± 12.2 years in the A-PRP group.	Unilateral hip pain with functional impairment for at least four months. Baseline pain intensity of at least 20 on a 100 mm VAS. BMI < 35. Failure of conservative treatment. Radiographic evidence of low or intermediate OA severity (Tonnis grades 1 to 3).	Age under 18 or over 65. Inability to provide informed consent. Systemic disorders, including cardiovascular disease, infections, and immune system disorders. Neoplastic conditions or local infections. OA secondary to hip dysplasia, collapse deformity, osteonecrosis, Perthes disease, or epiphysiolysis. Patients with inflammatory arthritis, such as rheumatoid arthritis. Local skin lesions at the injection site. Pregnancy. Platelet count <150,000/mm³ or hemoglobin <11 g/dL at blood harvest.

**Table 2:** Diagnostic criteria and approaches for intra-articular injections OA: osteoarthritis; AP: anteroposterior; ASA: amniotic suspension allograft; SVF: stromal vascular fraction; BM-MSCs: bone marrow-derived mesenchymal stem cells; AD-MSCs: adipose-derived mesenchymal stem cells; MFAT: microfragmented adipose tissue; PRP: platelet-rich plasma; BMC: bone marrow concentrate; KL: Kellgren-Lawrence; OHS: Oxford Hip Score; GMP: good manufacturing practice; MSC: mesenchymal stem cell

Study	Methods	
Author, Year, Country, Design	Diagnosis criteria	Intra-articular injections
Meadows et al. [15], 2021, United States, Prospective Case Series	Criteria for diagnosing hip OA: Radiographic assessment using AP pelvis radiographs to classify OA severity into Tonnis grades 1 and 2.	Single intra-articular injection of 2 mL of ASA mixed with 0.9% saline (final volume 4 mL). Guided by ultrasound imaging. Effusions were aspirated to prevent dilution of the injected ASA.
Onoi et al. [16], 2023, Japan, Prospective Case Series	KL classification based on radiographic findings. Patients with KL grades II, III, and IV were included in the study.	SVF cells were obtained from subcutaneous fat (abdomen or breech) using the Celution® 800/CRS system. The harvested adipose tissue (120-350 mL) was processed to obtain SVF cells, which were concentrated, washed, and prepared in a final volume of 5 mL of lactated Ringer's solution. At least 2.5 × 10⁷ SVF cells were injected intra-articularly per hip joint using a catheterized needle under ultrasound guidance, without anesthesia.
Heidari et al. [21], 2022, United Kingdom, Observational, Intention-to-treat Comparative	KL grading system based on radiographic assessment.	Adipose tissue was harvested from the patient’s subcutaneous fat using a Lipogems® system. The processed MFAT was injected under ultrasound guidance into the hip joint. PRP was prepared using Endoret® (prgf®) technology, and patients in the MFAT + PRP group received 4 mL of MFAT mixed with 2 mL of PRP. All patients followed a standardized physiotherapy protocol post-injection.
Rodriguez-Fontan et al. [22], 2018, United States, Prospective Cohort	Radiographic assessment using anteroposterior and lateral X-rays of the hip joint.	Bone marrow aspirate was harvested from the anterior iliac crest using a bone marrow aspiration needle. Approximately 120 mL of bone marrow was collected and centrifuged at 1400 g for 15 minutes to concentrate MSCs and growth factors. The final BMC volume of 12 mL was injected into the hip joint under radiographic or ultrasound guidance.
Natali et al. [17], 2023, Italy, Prospective Case Series	Radiographic assessment using the KL classification system. Patients were also classified according to the OHS into early, moderate, moderate-to-severe, and severe OA categories.	The Lipogems ortho kit was used to obtain MFAT. Patients received a 4 mL intra-articular injection of autologous MFAT under ultrasound guidance. Post-treatment protocol included three days of crutch use, antithrombotic prophylaxis, and application of an elastic waistband at the harvesting site for 15 days.
Mardones et al. [23], 2017, Chile, Prospective Cohort Study	Radiographic evaluation of osteoarthritis severity using the Tonnis classification system.	Bone marrow aspirate (30 mL for unilateral treatment, 60 mL for bilateral) was collected from the posterior iliac crest. The aspirate was processed to isolate and expand BM-MSCs in a GMP facility. Patients received three intra-articular injections of 20 million BM-MSCs per hip, administered over three consecutive weeks.
Dall’Oca et al. [18], 2019, Retrospective Case Series	Clinical examination and radiographic evaluation using the Tonnis grading system.	The abdominal wall was selected as the donor site for adipose tissue harvesting. Local anesthesia (Klein solution) was injected before adipose tissue extraction. Approximately 60 cc of adipose tissue was harvested and processed using the Lipogems system to obtain microfragmented adipose tissue rich in MSC. Between 5 and 10 mL of the processed material was injected intra-articularly under fluoroscopic guidance while the affected limb was under traction.
Burnham et al. [19], 2021, Canada, Prospective Cohort	Radiographic assessment using the KL classification system.	Bone marrow aspirate was collected from the posterior ilium (20–30 mL per joint injected). Bone marrow was processed via centrifugation at 1000 × g for five minutes to concentrate MSCs and other beneficial cells. A total of 8–10 mL of BMC was injected into each joint under ultrasound guidance. For hip injections, an anterior synovial recess approach was used.
Mazzotta et al. [20], 2022 Italy, Observational, Intention-to-Treat Comparative	Radiographic assessment using the Tonnis classification system.	Autologous PRP was prepared by collecting 150 mL of autologous blood, centrifuging at 1800 rpm for 15 minutes, and then processing to obtain a final platelet-rich plasma volume of 20 mL. Umbilical cord PRP was derived from pooled cord blood donations, processed following the guidelines of the Italian regulatory framework, and concentrated via centrifugation. PRP formulations were activated by adding 10% calcium gluconate before injection. All injections were performed using ultrasound guidance. Patients received a total of three intra-articular injections at one-week intervals.

**Table 3:** Methods for outcome measurements and time of follow-up OA: osteoarthritis; AP: anteroposterior; ASA: amniotic suspension allograft; VAS: visual analog scale; HHS: Harris Hip Score; mHHS: modified Harris Hip Score; iHOT-12: International Hip Outcome Tool–12; SANE: Single Assessment Numerical Evaluation; SF-12: 12-Item Short Form Health Survey; JHEQ: Japanese Orthopaedic Association Hip Disease Evaluation Questionnaire; MRI: magnetic resonance imaging; OHS: Oxford Hip Score; WOMAC: Western Ontario and McMaster Universities Arthritis Index; PDQQ: Pain Disability Quality of Life Questionnaire

Study	Results		
Author, Year, Country, Design	General outcomes	Imaging outcomes	Follow-up
Meadows et al. [15], 2021, United States, Prospective Case Series	mHHS; iHOT-12; VAS; SANE; SF-12	AP pelvis and lateral hip radiographs at baseline and 12 months post-injection. Joint space narrowing, osteophytes, cysts, and subchondral sclerosis were assessed.	Patients were assessed at baseline, 1 week, 1 month, 2 months, 3 months, 6 months, and 12 months.
Onoi et al. [16], 2023, Japan, Prospective Case Series	HHS; JHEQ; VAS	Assessed changes in the center-edge angle and acetabular head index T2 mapping MRI: Used to analyze cartilage integrity before and after treatment.	Clinical assessments were performed at baseline, 1 month, 3 months, and 6 months post-injection. Imaging assessments were conducted preoperatively and at 6 months post-injection.
Heidari et al. [21], 2022, United Kingdom, Observational, Intention-to-treat Comparative	VAS; OHS. Patients were categorized as non-responders, responders, or super-responders based on changes in VAS and OHS.	Follow-up imaging not performed.	Clinical assessments were conducted at baseline, 3 months, 6 months, and 12 months post-injection.
Rodriguez-Fontan et al. [22], 2018, United States, Prospective Cohort	WOMAC. The Minimal Clinically Important Difference for WOMAC was set at 9.15 points.	Follow-up imaging not performed.	Patients were assessed at 6-, 12-, 18-, and 24-month post-injection.
Natali et al. [17], 2023, Italy, Prospective Case Series	OHS; VAS	AP pelvis and lateral hip radiographs at baseline and 29-41 months post-injection. Joint space narrowing, osteophytes, cysts, and subchondral sclerosis were evaluated using the Kellgren-Lawrence classification.	Clinical evaluations occurred between 29 and 41 months after the initial injection.
Mardones et al. [23], 2017, Chile, Prospective Cohort Study	HHS; VAS; WOMAC; Vail Hip Score	Radiographic changes were evaluated using the Tonnis classification.	Patients were followed for 16 to 40 months after treatment.
Dall’Oca et al. [18], 2019, Retrospective Case Series	HHS; WOMAC; VAS	Follow-up imaging not performed.	Clinical and functional assessments were performed preoperatively and at six months post-injection.
Burnham et al. [19], 2021, Canada, Prospective Cohort	PDQQ; VAS Patients were categorized as responders if they achieved ≥50% pain relief and ≥50% improvement in PDQQ scores at six months.	Follow-up imaging not performed.	Patients were assessed at baseline, three months, six months, and 12 months post-injection.
Mazzotta et al. [20], 2022 Italy, Observational, Intention-to-Treat Comparative	HHS; WOMAC; VAS	Follow-up imaging not performed.	Patients were assessed at baseline, 2 months, 6 months, and 12 months post-injection.

**Table 4:** Findings, limitations, and risk of bias OA: osteoarthritis; HHS: Harris Hip Score; mHHS: modified Harris Hip Score; iHOT-12: International Hip Outcome Tool–12; SANE: Single Assessment Numerical Evaluation; VAS: visual analog scale; JHEQ: Japanese Orthopaedic Association Hip Disease Evaluation Questionnaire; KL: Kellgren-Lawrence; OHS: Oxford Hip Score; WOMAC: Western Ontario and McMaster Universities Arthritis Index; PDQQ: Pain Disability Quality of Life Questionnaire; C-PRP: umbilical cord platelet-rich plasma; A-PRP: autologous platelet-rich plasma; BMC: bone marrow concentrate; MCID: Minimal Clinically Important Difference

Study	Results		
Author, Year, Country, Design	Findings	Adverse effects	Limitations and Risk of Bias
Meadows et al. [15], 2021, United States, Prospective Case Series	Nine patients completed the study (one withdrew and underwent total hip arthroplasty). Pain and function improvement were statistically significant at 6 and 12 months. iHOT-12 scores improved significantly from baseline to 12 months (P = 0.02). SANE scores improved significantly from baseline to 6 months (P < 0.01) and 12 months (P < 0.01). mHHS scores improved significantly from baseline to 6 months (P = 0.02) and 12 months (P = 0.01).	No major adverse events were reported. Two patients showed a transient increase in CRP levels, but no clinical symptoms were observed, and values normalized by 6 months. One patient withdrew at 2 months and underwent total hip arthroplasty. No significant changes in joint space narrowing or radiographic markers at 12 months.	Very small sample size, in addition to attrition bias. Single-arm study, making it impossible to separate the placebo effect/regression to the mean. Limited follow-up (short- to medium-term). Outcomes focused on clinical measures/self-report. Little objective evidence of structural regeneration. Injection protocol not fully described to a level that allows easy replication by the reader.
Onoi et al. [16], 2023, Japan, Prospective Case Series	Significant improvement in clinical scores at 1-, 3-, and 6-month post-injection. HHS increased from 25.2 to 46.8 at 6 months. JHEQ scores improved significantly across all subscales (pain, movement, mental health). VAS pain score decreased from 75.5 to 46.5 at 6 months. Patients with KL grade II showed the best improvement, while KL grade IV patients had only slight improvements. No significant changes in radiographic parameters. No significant improvement in T2 mapping MRI values, suggesting no major structural cartilage regeneration within 6 months.	No severe adverse events occurred. Five patients (11.9%) reported mild hip pain for several days post-injection, but all cases resolved within a week. No infections or need for total hip replacement were observed during the follow-up period.	Short follow-up. Heterogeneous population (KL II–IV). High risk of confounding bias.
Heidari et al. [21], 2022, United Kingdom, Observational, Intention-to-treat Comparative	Both treatment groups showed significant improvements in pain and function over 12 months. VAS scores decreased by 20.8 points in the MFAT group and 12.8 points in the MFAT + PRP group. OHS improved by 6.57 points in the MFAT group and 7.34 points in the MFAT + PRP group. Super-responders (patients with ≥20-point improvement in VAS) were 64% in the MFAT and 63% in the MFAT + PRP groups. No significant difference between the two treatments in terms of pain relief and functional improvement. Twenty patients (10 from each group) eventually required total hip arthroplasty due to lack of response to treatment.	No infections or thromboembolic events. Some patients experienced mild joint pain post-injection, which resolved within a few days.	Attrition bias (~14–18% lost-to-follow-up). Conflicts of interest declared by some authors (holdings in related companies). Significant heterogeneity in OA severity. Lack of standardized description of cell/product quantification among patients. High risk of confounding bias.
Rodriguez-Fontan et al. [22], 2018, United States, Prospective Cohort	WOMAC scores improved significantly from baseline (40.8%) to final follow-up (20.6%) (P <.001 satisfaction of patients reported being satisfied with the treatment. mcid threshold was reached by patients. two required total hip arthroplasty within months due to treatment failure rate)	No major complications were observed. Common minor side effects included: - Hip joint discomfort in the first few days after injection (36.8%). - Mild pain at the BMC extraction site (15.8%). - Pain during the first two weeks post-injection (26.3%).	Small sample. Inclusion criteria limited to early OA. Short median follow-up. High risk of confounding bias.
Natali et al. [17], 2023, Italy, Prospective Case Series	Among the 55 patients included in the final analysis, 28 did not require additional treatments, with their OHS improving by an average of 6.9 points. Ten patients underwent a second intra-articular injection, with a mean interval of 635.7 days between injections. Seventeen patients eventually required total hip replacement, with a mean delay of 495 days after MFAT injection. Patients with early and moderate osteoarthritis showed the greatest improvement, while those with severe osteoarthritis were more likely to progress to surgery. No significant radiographic improvements were observed, although VAS pain scores decreased from 4.7 to 1.8 over the follow-up period.	Only one patient experienced an adverse event (a deep bruise at the harvest site, which resolved over a few months).	Attrition bias (16 lost to follow-up). High conversion rate to arthroplasty during follow-up. Very high heterogeneity in OA severity without subgroup analysis. High risk of confounding bias.
Mardones et al. [23], 2017, Chile, Prospective Cohort Study	Significant improvement was observed in pain, function, and range of motion. VAS scores decreased from 4.2 to 1.1 (P = 0.0001). WOMAC scores improved from 34.5 to 19.2 (P = 0.15). HHS improved from 61.9 to 85.7 (P = 0.003). Vail Hip Score improved from 61.2 to 78.2 (P = 0.02). Nine out of ten patients showed no radiographic progression of osteoarthritis.	No complications, infections, or severe adverse events were reported.	Very small sample size. Laborious protocol, resulting in logistics and costs that limit replicability in a routine clinical setting. Short follow-up period. High risk of confounding bias.
Dall’Oca et al. [18], 2019, Retrospective Case Series	HHS improved from a baseline mean of 67.2 ± 3.4 to 84.6 ± 6.3 at six months (P = 0.0001). WOMAC scores decreased from 36.3 ± 4.7 at baseline to 19.8 ± 3.4 at six months (P = 0.0001). VAS scores improved from 4.6 ± 0.8 preoperatively to 1.5 ± 0.5 at six months (P = 0.0001). No patient showed worsening of their preoperative condition.	No major complications were reported. One patient experienced a minor organized hematoma at the adipose tissue harvesting site.	Very small sample size. Short follow-up. Inclusion criteria limited to early OA. High risk of confounding bias.
Burnham et al. [19], 2021, Canada, Prospective Cohort	Significant pain reduction and functional improvement were observed at all follow-up time points. Pain scores decreased by 46% at three months, 57% at six months, and 39% at 12 months post-injection. At six months, 70% of patients were classified as responders. Patients receiving hip injections showed less pain relief compared to knee injections. Seven patients required joint arthroplasty within one year (three knee and four hip cases).	Two patients developed acute synovitis within three days of injection, but no infections were reported. No major complications occurred.	Results generally presented in aggregate form (knee + hip), being impossible to reliably extract the outcome for hips alone. Limited follow-up. Heterogeneity in bone marrow concentrate preparation/quality and possible technical variations between centers/operators. High risk of confounding bias.
Mazzotta et al. [20], 2022 Italy, Observational, Intention-to-Treat Comparative	Both groups showed mild improvements in pain and function, but no significant long-term benefits. At two months, C-PRP resulted in a significant improvement in VAS (p = 0.031) and HHS (p = 0.011) compared to baseline. At 12 months, patients with lower osteoarthritis severity (Tonnis grades 1-2) showed better outcomes with C-PRP than A-PRP in terms of HHS improvement (p = 0.049). No significant differences were observed between A-PRP and C-PRP in the overall patient population.	Five patients in the A-PRP group (10.0%) and two in the C-PRP group (4.3%) reported mild pain after injection, which resolved within a few days.	Comparative study without a placebo-controlled group (possibility of allocation/expectation bias). Lack of use of “stem cell therapy” in the classical sense (C-PRP, with perinatal components) - affecting comparability with the other studies. Short follow-up to assess structural changes. Heterogeneity in product preparation methods and injection protocols (number of applications/volume).

**Figure 2:**
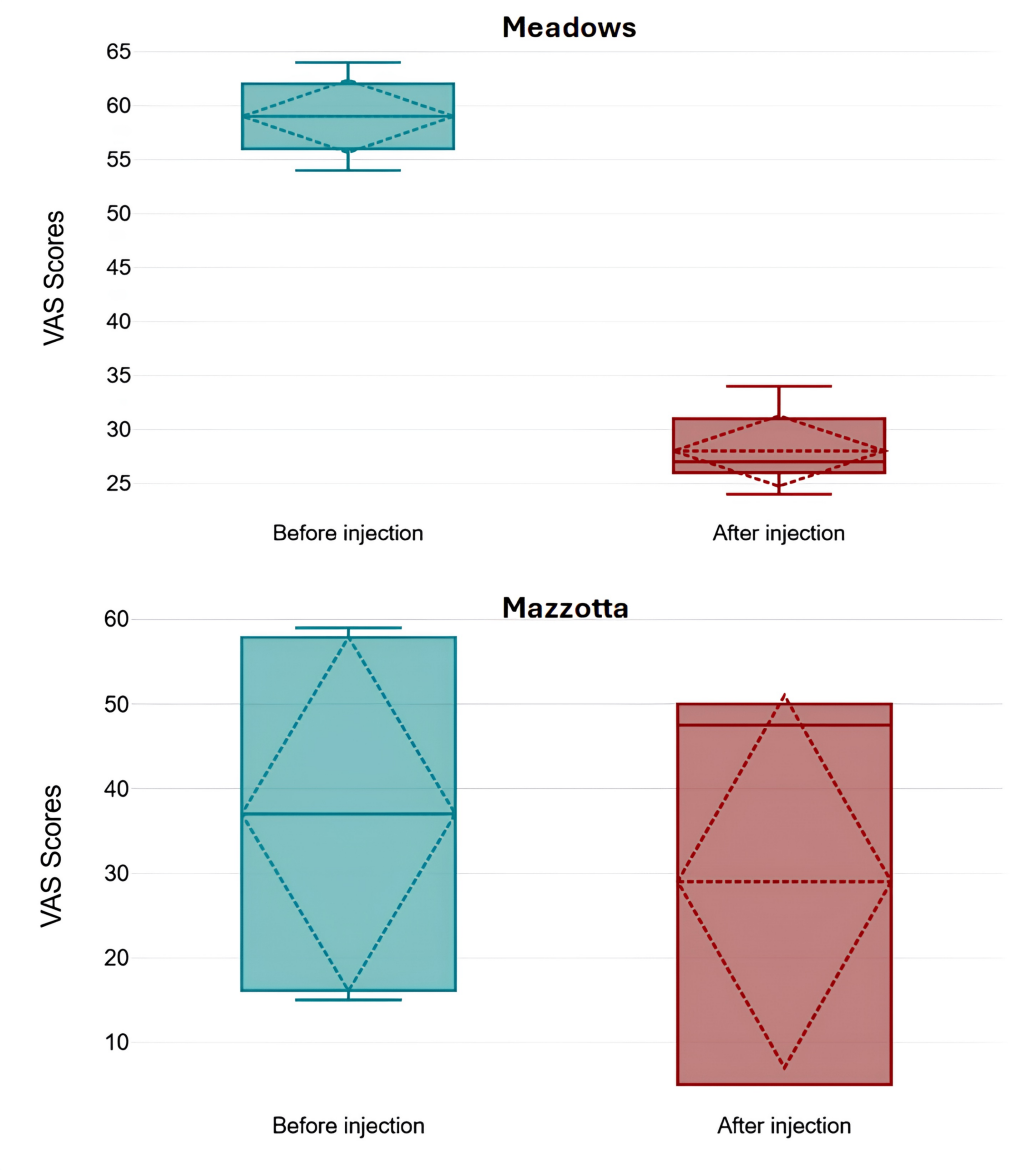
Mean, standard deviation, median, and interquartile range of VAS scores before and after 12 months from of treatment with perinatal-related MSC. Only the results from Meadows et al. were statistically significant. Data extracted from the studies Meadows et al. (2022) and Mazzotta et al. (2022) [15,20]. VAS: visual analog scale; MSCs: mesenchymal stem cells

**Figure 3:**
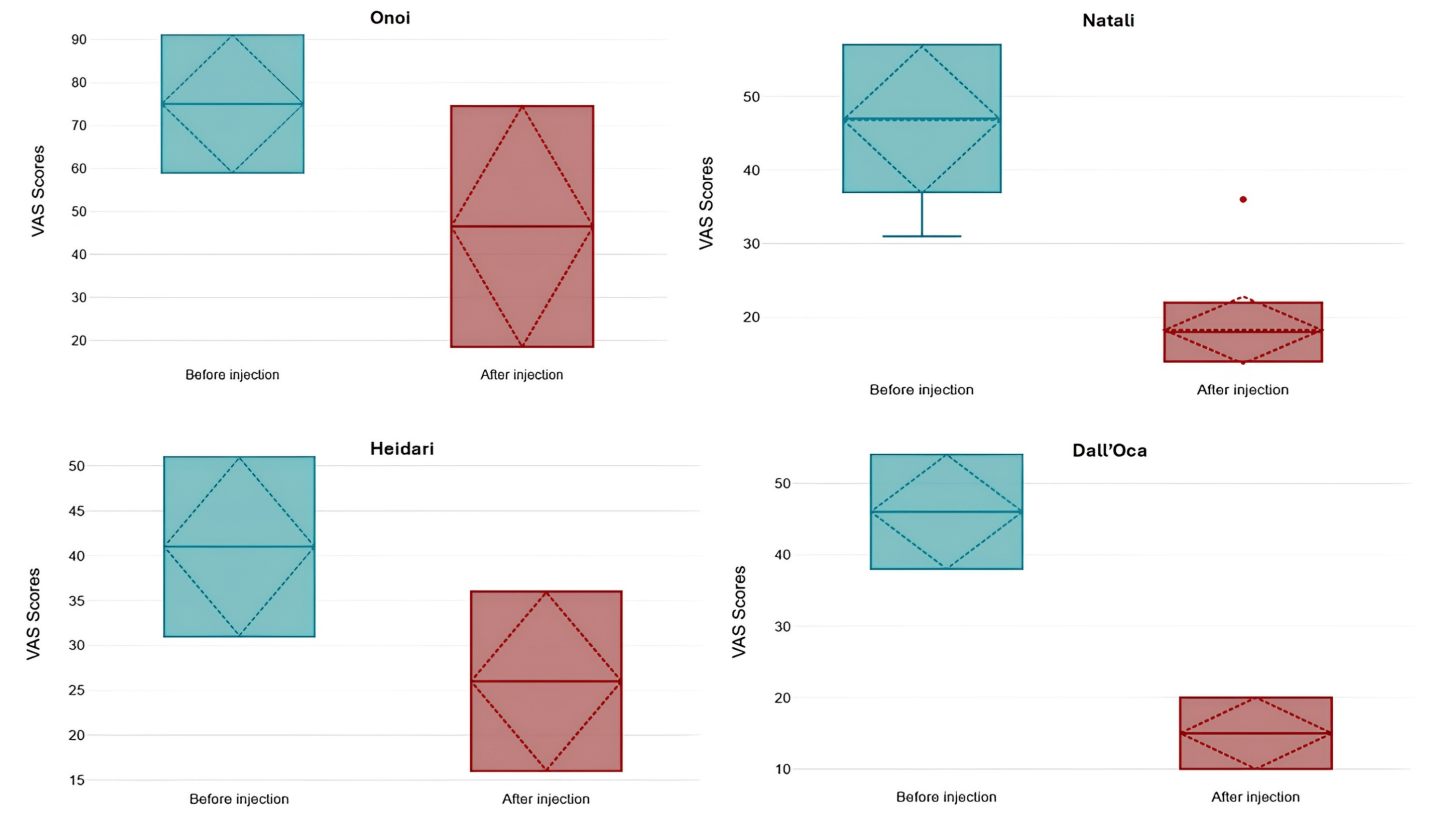
Mean, standard deviation, median, and interquartile range of VAS scores before and after 6 (Dall’oca and Onoi), 12 (Heidari), and >29 (Natali) months from treatment with AD-MSC. All four results were statistically significant. Data extracted from the studies Onoi et al. (2023), Natali et al. (2023), Dall’Oca et al. (2019), and Heidari et al. (2022) [16-18,21]. AD-MScs: Adipose-derived mesenchymal stem cells; VAS: visual analog scale

**Figure 4:**
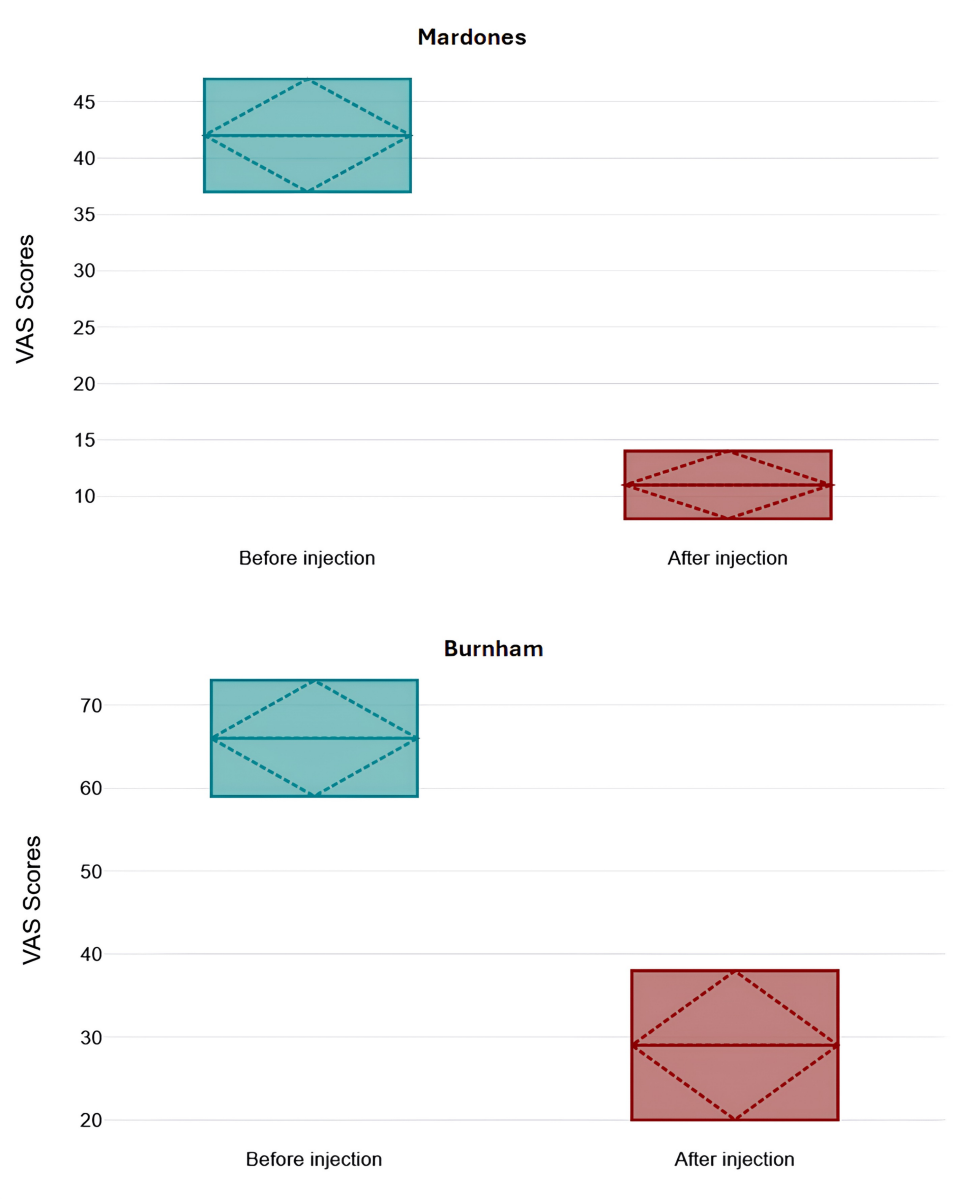
Mean, standard deviation, median, and interquartile range of VAS scores before and after 6 (Burnham) and >16 (Mardones) months from treatment with BM-MSC. Both results were statistically significant. Data extracted from studies Mardones et al. (2015) and Burnham et al. (2021) [19,23]. BM-MSCs: bone marrow-derived mesenchymal stem cells; VAS: visual analog scale

All studies demonstrated significant improvements in pain and joint function through the use of various stem cell-based therapies. Pain reduction, commonly measured using the visual analog scale (VAS), showed an average decrease ranging between 30% and 50% in multiple studies [15,16,20-23]. One study reported that VAS scores dropped from 75.5 to 46.5 over six months following SVF cell therapy [16]. Similarly, another found a reduction in pain intensity from 4.2 to 1.1 in patients treated with bone marrow aspirate concentrate injections, showing substantial pain relief over time [22]. Microfragmented adipose tissue injections alone resulted in a 20.8-point reduction in pain scores, while their combination with PRP achieved a 12.8-point decrease over 12 months [21]. These findings illustrate consistent pain relief across different therapeutic approaches. Figures 2-4 quantitatively summarize VAS score changes before and after treatment for each type of MSC (perinatal-related MSC, AD-MSC, and BM-MSC) - combining studies according to cell type.

Functional outcomes improved significantly in most cases, as measured by the Harris Hip Score (HHS) and the Western Ontario and McMaster Universities Osteoarthritis Index (WOMAC) [15,18,19,21]. One study showed that HHS scores rose from a baseline of 61.9 to 85.7 following bone marrow-derived mesenchymal stem cell injections, reflecting marked gains in joint function and mobility [23]. Another reported a reduction in WOMAC scores from 36.3 to 19.8 at six months after intra-articular injections of adipose-derived mesenchymal stem cells, highlighting improvements in pain, stiffness, and physical function [18]. Additionally, improvements in quality of life and overall function were evident in patients receiving bone marrow concentrate therapy, with 70% achieving at least a 50% improvement in key functional metrics [19]. Table 3 shows the main results and outcomes from the studies.

Radiological evaluations were performed in some studies, yielding mixed results. One investigation found no significant radiological changes or evidence of cartilage regeneration following microfragmented adipose tissue injections, despite clinical improvements [21]. Conversely, another study using adipose-derived mesenchymal stem cell therapy reported cartilage repair in 60% of participants, as confirmed by MRI, suggesting the potential for structural benefits in certain cases [18].

The safety profiles of the therapies were generally favorable, with no severe adverse events reported across the studies [15,16,21-23]. Minor side effects included temporary joint discomfort, mild erythema, and swelling at injection sites. One study reported minor bruising at the adipose tissue harvest site, while another noted a small hematoma that resolved without intervention [17,18]. Inflammatory markers such as CRP were temporarily elevated in some cases but normalized without clinical complications [15]. Overall, the treatments were well-tolerated, with no infections, thromboembolic events, or long-term complications observed, underscoring their safety and potential viability for treating hip osteoarthritis.

Discussion

Stem cell-based therapies for hip OA consistently demonstrated symptomatic relief, though the magnitude of improvement varied across studies. Pain reduction and functional gains have been reported for amniotic suspension allograft and BM-MSCs, yet long-term evidence of cartilage regeneration currently remains limited [15,22]. Interestingly, AD-MSCs offered comparable clinical improvements [16,21]. We also showed that combining PRP with MSCs did not significantly enhance outcomes, suggesting that immunomodulatory mechanisms, rather than direct regenerative effects, may be the primary driver of symptomatic relief [21].

The choice of stem cell source does not appear to be among the most relevant factors for predicting outcomes. At the same time, BM-MSCs possess strong chondrogenic potential and have been historically regarded as the gold standard [22]. However, invasive extraction procedures and limited cell yield reduce their practicality for routine clinical use. AD-MSCs provide a readily accessible and abundant alternative with less invasive harvesting techniques, demonstrating promising equivalent clinical efficacy [16,21]. PRP-based approaches, including umbilical cord-derived PRP (C-PRP), have shown early improvements in pain and function, and differences between C-PRP and autologous PRP appear minimal [20]. These findings highlight that practicality can be taken into account when choosing the cell source.

The injection strategy can significantly influence therapeutic outcomes. Single-dose administrations, as used in some studies [15,19], provide immediate symptomatic relief but may lack durability. Multi-dose protocols, such as repeated BM-MSC injections over several weeks, can result in sustained functional improvement and stable imaging findings, although the sample size was small in the only study that was done [23]. Patient-specific factors, including OA severity and joint biomechanics, may also impact efficacy, as hip OA appears less responsive to BM-MSCs than knee OA [19]. Future studies need to standardize delivery protocols, including cell dose, frequency, and administration technique, as it remains a key challenge for clinical translation.

Baseline osteoarthritis severity appeared to modulate treatment response across the included studies. In a microfragmented adipose tissue (MFAT) cohort including KL 1-4 hips, most patients who later required THA were KL 3-4, suggesting poorer outcomes in advanced disease [21]. Similarly, a three-year MFAT series stratified by baseline Oxford Hip Score showed that early and moderate OA achieved more durable benefits and had the lowest THR rates, whereas moderate-to-severe and severe groups accounted for most arthroplasties [17]. Studies restricted to milder radiographic grades (e.g., Tönnis 1-2) reported symptomatic improvement but no consistent structural regeneration, reinforcing better responsiveness in less advanced disease [15,22,23]. Nonetheless, improvement was still observed in mixed-severity SVF series, though higher-grade joints again showed greater conversion to THR [16,17,21].

Few studies prespecified age-stratified response analyses. In a Canadian BMC cohort including KL 1-4 hips, outcome improvements at 6 months were not associated with patient age or radiographic severity [19]. Likewise, in a-PRP comparator trial included in our evidence base, age did not significantly affect 12-month outcomes, whereas Tönnis grade did, with lower-grade hips demonstrating superior responses [20]. Collectively, the available evidence indicates that baseline disease severity (i.e., milder Tönnis/KL grades or “early-moderate” OHS categories) represents a more reliable predictor of therapeutic responsiveness than chronological age alone [17,19-22].

Stem cell-based interventions for hip OA exhibited a generally favorable safety profile. Most adverse events were mild and transient, including injection-site pain or swelling [16,21]. Rare events, such as acute synovitis or potential immune reactions, particularly with allogeneic sources, underscore the importance of monitoring and standardized procedures [15,19]. No serious infections or thromboembolic complications were reported across studies, supporting the relative safety of these interventions when performed according to current protocols [20].

Limitations

While the results of the included studies are encouraging, several limitations must be highlighted. First, the small number of available clinical studies (n = 9), most of which have small sample sizes and lack randomization or control groups, restricts the strength of conclusions and underlines how early this field still is. Second, the use of heterogeneous and often non-standardized outcome measures, particularly the reliance on VAS, with less frequent reporting of validated functional scores such as HHS and WOMAC, limits comparability across trials. Third, protocols varied widely in terms of stem cell source, preparation, dosage, number of injections, and imaging guidance. This lack of standardization significantly hampers reproducibility and clinical translation. Additionally, radiological and MRI outcomes remain inconsistent, with no reliable evidence of structural cartilage regeneration, suggesting that current benefits may largely derive from anti-inflammatory or paracrine effects rather than true regeneration. Follow-up periods were generally short (ranging from 6 to 36 months), precluding conclusions about long-term efficacy or durability of stem cell-based therapies. These limitations highlight the preliminary nature of the available evidence.

While preliminary results suggest that stem cell-based therapies for hip OA may provide symptomatic relief and improved function, the evidence remains insufficient to guide routine clinical use. Given the lack of standardization in protocols, absence of RCTs, and short-term follow-up, these interventions should currently be restricted to research settings. Clinicians and patients should be aware that stem cell therapies are still investigational, and robust, high-quality trials are required before their widespread adoption can be recommended.

Future research must address these gaps with well-designed, multicenter randomized controlled trials that include larger patient populations, longer follow-up periods, and rigorous safety monitoring. Standardization is essential, particularly regarding stem cell sourcing, processing, dosing, and delivery techniques, to enable reproducibility and facilitate regulatory approval. Equally important is the consistent use of validated functional scales and advanced imaging modalities, moving beyond VAS alone, to capture clinically meaningful outcomes. Identifying patient subgroups most likely to benefit (e.g., by OA severity, metabolic status, or baseline biomarkers) will support personalized approaches. Finally, cost-effectiveness analyses and health policy research are needed to determine whether stem cell therapies can realistically be integrated into routine clinical practice.

On the other hand, the cost-effectiveness and feasibility of large-scale implementation of MSC therapies remain unresolved. A recent study demonstrated that a type of MSC therapy can be cost-effective compared with surgical microfracture, with incremental cost-effectiveness ratios below common willingness-to-pay thresholds [24]. However, real-world adoption is challenged by the absence of standardized quality-control processes and the proliferation of unregulated clinics offering stem cell treatments without sufficient oversight [25]. These issues raise ethical, safety, and regulatory concerns that may hinder patient access and delay integration into standard practice. Therefore, long-term economic evaluations and health policy research are also essential to confirm the viability of these therapies, ensuring a sustainable implementation.

## Conclusions

From a clinical perspective, stem cell therapy emerges as a promising but still experimental approach for hip osteoarthritis. The current evidence consistently shows short- to mid-term benefits in pain relief and functional improvement, yet three critical limitations must be emphasized. At first, the lack of structural regeneration evidence: radiological findings remain inconsistent, with no robust proof of cartilage regeneration. Secondly, there is a significant heterogeneity of protocols. Wide variability in cell source, dosage, number of injections, and guidance techniques prevents reproducibility and limits clinical translation. And finally, outcomes are not standardized. Most studies rely on VAS, or very heterogeneous functional scales, while validated functional scales and imaging outcomes are underreported. Based on these findings, our review delivers a clear message: stem cell-based injections for hip OA should currently be regarded as experimental, safe, and symptom-modifying interventions rather than established regenerative treatments.

## Appendices

Search strategies for each database

PUBMED

 ("osteoarthritis, hip"[MeSH Terms] OR "Hip Osteoarthritis"[Title/Abstract] OR "Coxarthrosis"[Title/Abstract] OR "Coxarthroses"[Title/Abstract] OR "osteoarthritis of hip"[Title/Abstract]) AND ("injections, intra articular"[MeSH Terms] OR "intra articular injection"[All Fields] OR "intra articular injection"[All Fields] OR "intraarticular injection"[All Fields] OR "intra articular"[All Fields] OR "intra articular"[All Fields] OR "Intraarticular"[All Fields] OR "injection"[All Fields] OR "Administration"[All Fields] OR "Infiltration"[All Fields]) AND ("Stem-Cells"[MeSH Terms] OR "stem cell"[Title/Abstract] OR "orthobiologic"[Title/Abstract] OR "cell-based"[Title/Abstract] OR "cell"[Title/Abstract] OR "biologic"[Title/Abstract] OR "adipose tissue"[Title/Abstract] OR "marrow"[Title/Abstract] OR "amniotic"[Title/Abstract] OR "amnio"[Title/Abstract])

"Osteoarthritis, Hip"[Mesh] OR "Hip Osteoarthritis"[tiab] OR "Coxarthrosis"[tiab] OR "Coxarthroses"[tiab] OR "Osteoarthritis Of Hip"[tiab]

(Intra-Articular NEXT Injection) OR (Intra Articular NEXT Injection) OR (Intraarticular NEXT Injection) OR "Intra-Articular" OR "Intra Articular" OR Intraarticular OR (Injection) OR Administration OR Infiltration

("Stem-Cells"[MeSH Terms] OR "stem cell"[Title/Abstract] OR "orthobiologic"[Title/Abstract] OR "cell-based"[Title/Abstract] OR "cell"[Title/Abstract] OR "biologic"[Title/Abstract] OR "adipose tissue"[Title/Abstract] OR "marrow"[Title/Abstract] OR "amniotic"[Title/Abstract] OR "amnio"[Title/Abstract])

EMBASE

('hip osteoarthritis'/exp OR 'hip osteoarthritis':ti,ab,kw OR 'coxarthrosis':ti,ab,kw OR 'coxarthroses':ti,ab,kw OR 'osteoarthritis of hip':ti,ab,kw) AND ('intraarticular drug administration'/exp OR 'intra articular injection':ti,ab,kw OR 'intraarticular injection':ti,ab,kw OR 'intra articular':ti,ab,kw OR 'intraarticular':ti,ab,kw OR 'injection':ti,ab,kw OR 'administration':ti,ab,kw OR 'infiltration':ti,ab,kw) AND ('stem cell'/exp OR 'stem cell':ti,ab,kw OR 'orthobiologic':ti,ab,kw OR 'cell-based':ti,ab,kw OR 'cell':ti,ab,kw OR 'biologic':ti,ab,kw OR 'adipose tissue':ti,ab,kw OR 'marrow':ti,ab,kw OR 'amniotic':ti,ab,kw OR 'amnio':ti,ab,kw)

Cochrane

1          MeSH descriptor: [Osteoarthritis, Hip] explode all trees      1392

2          ("Osteoarthritis, Hip" OR "Hip Osteoarthritis" OR "Coxarthrosis" OR "Coxarthroses" OR "Osteoarthritis Of Hip" OR "Osteoarthritis Of Hips"):ti,ab,kw        2523

3          1 OR 2 2523

4          MeSH descriptor: [Injections, Intra-Articular] explode all trees      1888

5          ((Intra-Articular NEXT Injection) OR (Intra Articular NEXT Injection) OR (Intraarticular NEXT Injection) OR "Intra-Articular" OR "Intra Articular" OR Intraarticular OR (Injection) OR Administration OR Infiltration):ti,ab,kw    125034

6          4 OR 5 125039

7          MeSH descriptor: [Stem Cells] explode all trees      1354

8          ((stem NEXT cell) OR (orthobiologic) OR "cell-based" OR (cell) OR (biologic) OR "adipose tissue" OR "marrow" OR "amniotic" OR "amnio"):ti,ab,kw            239484

9          7 OR 8 239502

10        3 AND 6 AND 9         26

SCOPUS

"Hip Osteoarthritis" OR "Coxarthrosis" OR Coxarthroses OR "Osteoarthritis Of Hip”

“Intra-Articular Injection” OR “Intra Articular Injection” OR “Intraarticular Injection” OR "Intra-Articular" OR "Intra Articular" OR Intraarticular OR Injection OR Administration OR Infiltration

"Stem-Cells" OR "stem cell” OR orthobiologic OR "cell-based" OR cell OR biologic OR "adipose tissue" OR marrow OR amniotic OR amnio

Web of Science

TS=("Hip Osteoarthritis" OR "Coxarthrosis" OR Coxarthroses OR "Osteoarthritis Of Hip”) AND TS=(“Intra-Articular Injection” OR “Intra Articular Injection” OR “Intraarticular Injection” OR "Intra-Articular" OR "Intra Articular" OR Intraarticular OR Injection OR Administration OR Infiltration) AND TS=("Stem-Cells" OR "stem cell” OR orthobiologic OR "cell-based" OR cell OR biologic OR "adipose tissue" OR marrow OR amniotic OR amnio)
